# Professional Secrets

**Published:** 1850-05

**Authors:** 


*Professional Secrets.*—In connection with the events of the trial for murder, recently concluded in the State of Massachusetts, which has created a deep interest throughout the country, the prisoner is stated in the newspaper prints, to have confessed to his physician, that, on his arrest, he took a grain of strychnia, which produced spasms, but failed to secure the intended object, which, it is presumed, was self-destruction. Without attaching any importance to any part of this statement, which, very likely, is nothing more than a street rumor, it may serve to suggest the subject of professional secrets. Were it true that an attempt at self-destruction had been made in this instance, and the fact communicated in professional intercourse with a physician in attendance, neither the Court nor the Public should have been any the wiser for the confession. It cannot be necessary to sustain this assertion by argument, but it is well for medical practitioners to keep before them their duty in such cases, so as not to be led astray through inadvertency.

It is plainly in accordance with propriety and morality, that professional secrets should be held sacredly confidential. Not only should they be not communicated for any sinister end, but a legal tribunal has no right to require them, even for the furtherance of the ends of justice. A person who, under the necessities of disease, is compelled to make revelations which might be used to his disadvantage, ought not to be exposed to danger therefrom, any more than he should be obliged to criminate himself in giving testimony. In the latter case, the law affords a just protection, by permitting him to withhold statements ; in the prior case, justice (on the same principle), demands protection from statements which a due regard to life and health forbids him to withhold from his medical adviser.

Inviolable secresy has ever been deemed both a right and duty appertaining to the medical profession. A solemn pledge to that effect was embraced in the celebrated Oath of Hippocrates. In this State, physicians are prohibited by express statute, from violation of professional confidence. The statute reads thus : "No person duly authorized to practice Physic or Surgery, will be allowed to disclose any information which he may have acquired in attending any patient in a professional character, and which information was necessary to enable him to prescribe for such patient as physician, or to do any act for him as a surgeon." N. Y. Revis. Stat., Vol. II., p. 406, Sec. 73. [Prof. Lee's notes to Guy's Forensic Medicine.]

The learned American editor of Guy's Forensic Medicine, states, that only in the States of Missouri and New York, is this prohibition made the subject of special statute. Of the legal enactments or practice in other States, we are not informed. In English Law, a medical man is bound to divulge secrets, when required to do so. Whatever may be the usage in this country, in States having no express laws on the subject, the Medical Practitioner, as it seems to us, is always honorably bound, by his duty to his patient, and to his Profession, to refuse to make public secrets, obtained by professional intercourse; and should he be required to deviate from this rule of conduct, it would, in our opinion, be not only justifiable, but incumbent on him, to incur the penalties of non-compliance, rather than sacrifice a cardinal principle of medical ethics, having a higher claim on his conscientious adherence, than the behests of any judicial authority.

